# Granzyme B PET Imaging of Combined Chemotherapy and Immune Checkpoint Inhibitor Therapy in Colon Cancer

**DOI:** 10.1007/s11307-021-01596-y

**Published:** 2021-03-12

**Authors:** Julian L Goggi, Siddesh V Hartimath, Tan Yun Xuan, Shivashankar Khanapur, Beverly Jieu, Hui Xian Chin, Boominathan Ramasamy, Peter Cheng, Tang Jun Rong, Yong Fui Fong, Tsz Ying Yuen, Rasha Msallam, Ann-Marie Chacko, Laurent Renia, Charles Johannes, You Yi Hwang, Edward G Robins

**Affiliations:** 1 Agency for Science, Technology and Research (A*STAR), Singapore Bioimaging Consortium, 11 Biopolis Way, #01-02 Helios, Singapore, 138667 Singapore; 2 Singapore Immunology Network, A*STAR, 8A Biomedical Grove, Immunos, Singapore, 138648 Singapore; 3 p53 Laboratory, A*STAR, 8A Biomedical Grove, #06-04/05, Neuros/Immunos, Singapore, 138665 Singapore; 4 Institute of Chemical and Engineering Sciences (ICES), A*STAR, 8 Biomedical Grove, #07, Neuros, Singapore, 138665 Singapore; 5 Laboratory for Translational and Molecular Imaging (LTMI), Cancer and Stem Cell Biology Programme, Duke-NUS Medical School, 8 College Road, Singapore, 169857 Singapore; 6 Clinical Imaging Research Centre (CIRC), Yong Loo Lin School of Medicine, National University of Singapore, Singapore, 117599 Singapore

**Keywords:** Tumor, Granzyme B, Lymphocytes, Chemotherapy, Checkpoint inhibitors

## Abstract

**Purpose:**

Chemotherapeutic adjuvants, such as oxaliplatin (OXA) and 5-fluorouracil (5-FU), that enhance the immune system, are being assessed as strategies to improve durable response rates when used in combination with immune checkpoint inhibitor (ICI) monotherapy in cancer patients. In this study, we explored granzyme B (GZB), released by tumor-associated immune cells, as a PET imaging-based stratification marker for successful combination therapy using a fluorine-18 (^18^F)-labelled GZB peptide ([^18^F]AlF-mNOTA-GZP).

**Methods:**

Using the immunocompetent CT26 syngeneic mouse model of colon cancer, we assessed the potential for [^18^F]AlF-mNOTA-GZP to stratify OXA/5-FU and ICI combination therapy response *via* GZB PET. *In vivo* tumor uptake of [^18^F]AlF-mNOTA-GZP in different treatment arms was quantified by PET, and linked to differences in tumor-associated immune cell populations defined by using multicolour flow cytometry.

**Results:**

[^18^F]AlF-mNOTA-GZP tumor uptake was able to clearly differentiate treatment responders from non-responders when stratified based on changes in tumor volume. Furthermore, [^18^F]AlF-mNOTA-GZP showed positive associations with changes in tumor-associated lymphocytes expressing GZB, namely GZB+ CD8+ T cells and GZB+ NK+ cells.

**Conclusions:**

[^18^F]AlF-mNOTA-GZP tumor uptake, driven by changes in immune cell populations expressing GZB, is able to stratify tumor response to chemotherapeutics combined with ICIs. Our results show that, while the immunomodulatory mode of action of the chemotherapies may be different, the ultimate mechanism of tumor lysis through release of Granzyme B is an accurate biomarker for treatment response.

**Supplementary Information:**

The online version contains supplementary material available at 10.1007/s11307-021-01596-y.

## Introduction

Tumors exploit immune checkpoint receptors to evade the immune system. Therapeutic immune checkpoint inhibitors (ICIs) activate lymphocytes, including T cells and NK cells to mount an effective immune response. Activated CD8 T cells and NK cells release Granzyme B leading to apoptosis of the tumor cells [1]. However, due to resistance and suppression mechanisms, the majority of patients do not show a durable response to ICIs [2–4]. Many combination clinical trials are currently underway driven by the hypothesis that cytotoxic chemotherapies may enhance responsiveness to ICIs by increasing tumor immunogenicity. Chemotherapeutics were originally thought to be immunosuppressive; however, recent studies show that many agents enhance antitumor effects by activating the immune system. Chemotherapies may promote tumor immunogenicity either by inducing immunogenic cell death as part of their therapeutic effect or by enhancing tumor antigen presentation or upregulating co-stimulatory molecules/downregulating co-inhibitory molecules expressed on the tumor cell surface, such as PDL-1 [5, 6]. 5-fluorouracil (5-FU) and oxaliplatin (OXA) are commonly used as first-line chemotherapeutics for the treatment of colorectal cancer [7] and have been shown to modulate the immune system [8–10]. Recent clinical trials have shown that chemotherapy combined with ICIs leads to an improvement in overall survival compared to ICI monotherapy alone (KEYNOTE trials 048, 189, 407) [11–13], potentially due to synergies in their immune-related mechanisms of action.

**Fig. 1.**
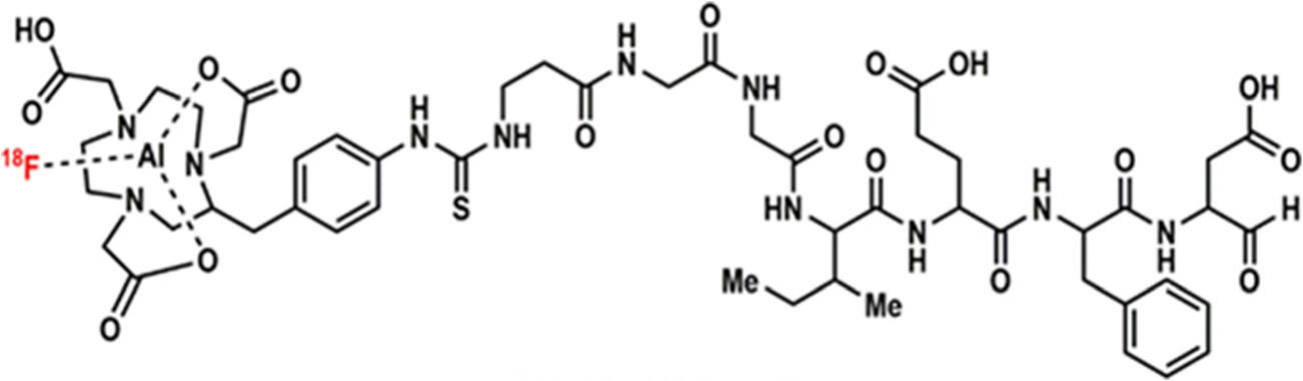
Structure of [^18^F]AlF-mNOTA-GZP.

Currently, there is a lack of specific biomarkers capable of providing a readout *in situ* of immune responses to different treatment strategies, complicating interpretation of clinical trials comparing different treatment strategies such as chemotherapy and ICIs. In the current study, we have assessed the ability of [^18^F]AlF-mNOTA-GZP (Fig. 1A), a peptide probe targeting granzyme B to serve as a PET imaging biomarker of combined ICI/chemotherapy in a syngeneic mouse model of colon cancer. PET imaging results were paired with multicolour flow cytometry analysis of the tumor-associated immune cell subsets for an in-depth comparative assessment of which immune cell types expressing GZB are best associated with tumor response.

## Materials and Methods

### General

H-Asp(OtBu)-H NovaSyn TG resin (0.21 mmol/g) was obtained from Merck. Fmoc-amino acids, HATU and HOAt were obtained from Advanced Chemtech. Fmoc-glutamic acid was t-butyl protected. (p-SCN-Bn)-NOTA was purchased from Boc Sciences and Macrocyclics. Glacial acetic acid was purchased from JT Baker. Sep-Pak® light (46 mg) Accell™ plus QMA carbonate cartridges and Sep-Pak® C18 light cartridges were purchased from Waters Corporation. Saline solution (0.9% w/v) was purchased from Braun Medical Industries. All other chemicals and reagents were purchased from Sigma-Aldrich, Fisher Scientific, and Tokyo Chemical Industry. No-carrier-added aqueous [^18^F]fluoride ion was produced *via* the [^18^O(p,n)^18^F] nuclear reaction (GE PETtrace 860 cyclotron). Quality control analytical radio-HPLC was performed on a UFLC Shimazdu HPLC system equipped with dual-wavelength UV detector and a NaI/PMT-radiodetector (Flow-Ram, LabLogic). Radioactivity measurements were made with a CRC-55tPET dose calibrator (Capintec, USA).

### [^18^F]AlF-mNOTA-GZP Radiochemistry

NOTA–β-Ala–Gly–Gly–Ile–Glu–Phe–Asp–CHO (mNOTA-GZP) was synthesized using standard Fmoc chemistry and characterized by HPLC and mass spectroscopy as previously described [14]. [^18^F]AlF-mNOTA-GZP was synthesized as previously described [15]. Briefly, aqueous [^18^F]fluoride was trapped on a preconditioned Sep-Pak® light (46 mg) Accell™ plus QMA carbonate cartridge, washed with water (5 mL), and eluted with 0.9% w/v saline (0.2 mL). To this was added glacial acetic acid (1 μL) to adjust to pH 4, 2 mM AlCl_3_ in 0.1 M pH 4 NaOAc buffer (24 μL) and ethanol (0.2 mL). This was then added into a reaction vial containing mNOTA-GZP (0.1 mg) and heated at 100 °C for 15 min. After cooling to room temperature, the crude reaction mixture was diluted with water (40 mL), loaded on a preconditioned Sep-Pak® C18 light cartridge and washed with water (5 mL). [^18^F]AlF-mNOTA-GZP was eluted with 70% ethanol in water (0.4 mL) and diluted with 0.9% w/v saline to a final concentration of 10% ethanol in saline. The radiochemical purity of [^18^F]AlF-mNOTA-GZP was assessed as previously described. [^18^F]AlF-mNOTA-GZP was obtained as a colorless solution in 10% ethanol in saline (pH=7.4) with a non-decay corrected radiochemical yield of 12-18% (total reaction and purification time 50 min), with a radiochemical purity of 98–99% and molar activity 37–62 GBq/μmol (*n*=5).

### Animal Procedures

All animal procedures were carried out following the Institutional Animal Care and Use Committee Singapore (IACUC No. 181399) and conformed to the US National Institutes of Health (NIH) guidelines and public law. BALB/c mice aged 6–8 weeks were purchased from In Vivos (Singapore). Mice were housed in specific-pathogen-free (SPF) environment during the experiments, at room temperature with a 12-h light-dark cycle and had free access to food and water.

The murine colon tumor cell line CT26 was acquired from ATCC and cultured in RPMI supplemented with 10% foetal bovine serum, 100 U/mL penicillin and 100 μg/mL streptomycin, at 37°C in a humidified atmosphere at 5% CO_2_. CT26 cells (2 × 10^5^) were prepared in a 1:1 (v:v) ratio in Matrigel (Sigma) and injected subcutaneously into the right shoulder of Balb/c mice. *In vivo* subcutaneous tumors were measured by callipers on days 6, 9, 12, 15, 19, and 21 after tumor inoculation. Tumor volume was then calculated using the modified ellipsoid formula 1/2(Length × Width^2^) [16].

Therapeutic immune checkpoint inhibitors (ICIs), chemotherapy, and combined ICI-chemo groups Rat IgG2a anti-mouse PD-1 (⍺PD1 mAb RMP1-14) and rat IgG2a isotype control (⍺-trinitrophenol mAb), were purchased from Bio-X Cell. All mice were treated by intraperitoneal (i.p.) injection of control IgG (5 mg/kg) or ⍺PD1 (10 mg/kg) on days 6, 9, and 12 following tumor inoculation. Oxaliplatin (OXA) and 5-fluorouracil (5-FU) were purchased from Sigma Aldrich. All mice were treated by intraperitoneal (i.p.) injection of saline, oxaliplatin (6.0 mg/kg, Q7D), 5-fluorouracil (70 mg/kg, Q3D) either alone or in combination with αPD1 as described.

In order to accurately assess tumor response to therapy tumor growth inhibition (%TGI) was determined using the formula %TGI = (V_c_-V_t_)/(V_c_-V_o_) × 100, where V_c_ and V_t_ are the mean tumor volumes of control and treated groups on day 21 and V_o_ is the tumor volume at the start of the study (Supplementary Table S3).

### PET-CT Imaging

Animals were imaged 14 days after tumor inoculation using a Siemens Inveon PET-CT. Briefly, animals were anesthetized using inhalational isoflurane (maintained at 1.5% alveolar concentration) and injected with [^18^F]AlF-mNOTA-GZP (~10MBq) via the lateral tail vein. Static PET acquisitions were performed at 60–80 min post-injection (p.i.) and CT scans were used for co-registration. Animals were monitored for maintenance of body temperature and respiration rate during imaging studies using the Biovet physiological monitoring system. Post-analysis of reconstructed calibrated images was performed with FIJI and Amide software (version 10.3 Sourceforge). Uptake of radioactivity in tissues was determined by the placement of volumes of interest (VOI) delineated by CT imaging. Data are expressed as % of the injected dose per gram (%ID/g) of tumor tissue in the VOI.

### Flow Cytometry

Tumors were excised immediately after *in vivo* PET imaging and freshly processed for flow cytometry. A single-cell suspension was generated by incubating in modified RPMI (Gibco) supplemented with 10% heat-inactivated fetal bovine serum (Gibco, Life Technologies), 20 μg/ml DNAse1 (Sigma-Aldrich) and 200 μg/ml Collagenase (Sigma-Aldrich). The samples were mechanically diced and incubated for 1 h at 37°C and dissociated into single cells by passing through a 100-μm cell strainer. The samples were then counted and assessed for viability with Trypan Blue (Sigma-Aldrich). Cells were stained with antibodies against CD45 (clone 30-F11 BV570; Biolegend), CD3 (clone 500A2 BUV563; BD Biosciences), CD4 (clone RM4-5 BV650; BD Biosciences), CD8 (clone 53-6.7 BV510; BD Biosciences), CD25 (clone PC61 BUV395; BD Biosciences), F4/80 (clone BM8 biotin; Biolegend), CD206 (clone C068C2 PE-Cy7; Biolegend), Ly6C (clone HK1.4 BV605; Biolegend), NKp46 (clone 29A1.4 BUV737; BD Biosciences), CD11b (clone M1/70 APC-Cy7; Biolegend), I-A/I-E (clone M5/114.15.2 BV785; Biolegend), Ly6G (clone 1A8 BV480; BD Biosciences), FoxP3 (clone 150D AlexaFluor647; Biolegend), Fixable Live/Dead Blue (Invitrogen), Streptavidin BUV805 (BD Biosciences), PD-L1 (clone MIH5 BV421; BD Biosciences), CD170 (clone E50-2440 PECF594; BD Biosciences), Perforin (clone S16009A PE; Biolegend), CD11c (clone N418 BV711, Biolegend), and Granzyme B (GZB clone QA16A02 AlexaFluor700; Biolegend). Flow cytometry was performed on a BD FACSymphony. Fluorophore compensations and detector voltage were set up using single stains on murine spleen cells. Data was recompensated and analyzed using FlowJo V10.7.1 software (FlowJo LLC).

### Dimension Reduction Analysis

Time-gated, size-gated, Live, singlet, CD45 positive cells from 46 fcs files were exported from FlowJo and used for dimension reduction analysis. t-Distributed Stochastic Neighbor Embedding (t-SNE) was used for unbiased dimension reduction and Rphenograph was used for clustering. t-SNE, clustering and overlay with t-SNE maps were performed with the cytofkit package in RStudio [17] (https://github.com/JinmiaoChenLab/cytofkit). The default cytofkit parameters were used for the analysis on 1000 cells from each fcs file, for a total of 46,000 cells. The following markers were used for the Rphenograph clustering: CD3, CD4, CD8, CD11b, CD11c, CD206, F4/80, Granzyme B, I-A/I-E, Ly6C, Ly6G, Nkp46 and Siglec-F.

### Statistical Analysis

Data were analyzed using a Kruskal Wallis 1-way ANOVA with a Dunn’s post-test using GraphPad Prism version 8.0.0 for Windows, GraphPad Software, San Diego, California USA, www.graphpad.com, *P*<0.05 was considered statistically significant. Data are expressed as mean ± S.D. unless otherwise indicated.

## Results

### Assessment of Treatment Efficacy Using [^18^F]AlF-mNOTA-GZP PET Imaging

**Fig. 2.**
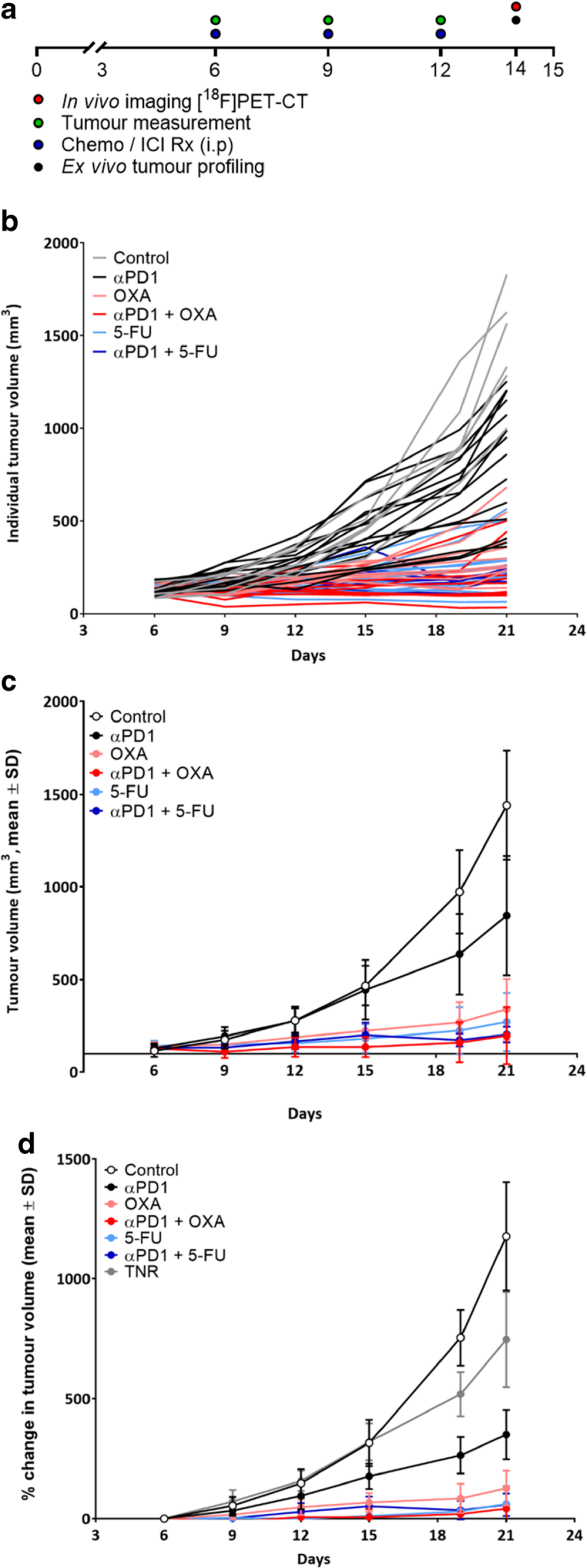
Comparison of therapeutic effect of chemotherapies, ICIs and combinations on change in tumor volume. A. Schematic representation of timeline shows dosing regimen. Mice (*n*=10–15) were i.p. treated with control IgG, αPD1, OXA, 5-FU or combinations of αPD1+OXA or αPD1+5-FU on days 6, 9, and 12 post tumor implantation. B. Individual tumor volume of CT26 tumor-bearing mice on days 6, 9, 12, 15, 19, and 21 post tumor implantation. C. Average tumor volume of CT26 tumor-bearing mice on days 6, 9, 12, 15, 19, and 21 post tumor implantation. Data are represented as mean ± S.D. D. Average tumor volume of CT26 tumor-bearing mice on days 6, 9, 12, 15, 19, and 21 post tumor implantation. Data are shown post separation of TNR group and represented as % change in tumor volume from the first day of assessment and are indicated as mean ± S.D. (TNR, treated non-responder).

Balb/c mice bearing CT26 colon tumors were treated with control IgG, ⍺PD1, OXA, 5-FU or a combination of ⍺PD1 and OXA or ⍺PD1 and 5-FU (Fig. 2A). Mouse tumor volumes were evaluated over time, with mice subjected to *in vivo* PET imaging at 14 days after tumor inoculation (8 days’ post-induction of therapy). We found that CT26 colon tumor growth curves were normally distributed (Shapiro-Wilk *p* 0.0615) and exhibited different responses to PD1 monotherapy, chemotherapy or combination chemo-ICIs based on tumor volumes (individual and grouped tumor volumes are shown in Fig. 2 and Table S1). Correlation analysis between %TGI and tumor uptake of [^18^F]AlF-mNOTA-GZP for all data without post hoc manipulation showed that they are well correlated (Pearson *r*=0.7139, ****p<0.0001, *n*=60). When the treatment arms were assessed individually, %TGI and tumor uptake of [^18^F]AlF-mNOTA-GZP were found to be correlated in the OXA treated arm (*r*=0.7325, *p<0.016, *n*=10), the combined αPD1&OXA treated arm (*r*=0.7396, *p<0.016, *n*=10), the 5-FU treated arm (*r*=0.7726, *p<0.0147, *n*=10) and the combined αPD1&5-FU treated arm (*r*=0.6741, *p<0.0326, *n*=10). However, correlation with the αPD1 monotherapy arm did not reach significance (*p*<0.0529, *n*=15) potentially due to the well documented variability in tumor response to αPD1 treatment.

Successful therapy response in this preclinical model was determined by the comparison of day 6 baseline tumor volumes with day 21 post therapy tumor volumes separating the treatment arms into two groups, treatment responders (combining complete responders and partial responders into a single group, TR) and treatment non-responders (TNR). This reductionist approach has been used previously to the assessment of the utility of imaging to stratify responders from non-responders but may introduce bias [15, 18–21]. TRs were identified as final tumor volumes less than 850 mm^3^ and include tumors with stable or decreased volumes (tumor volumes are shown in Supplementary Table S1). The volume of 850 mm3 was chosen as this is more than 2 standard deviations from the mean volume of the control group on day 21, using this approach there is only a 5% chance for a TR to be incorrectly assigned. Treatment response varied between treatment arms (Supplementary Table S2) with the combination treated groups exhibiting a greater response rate than the monotherapy groups.

**Fig. 3.**
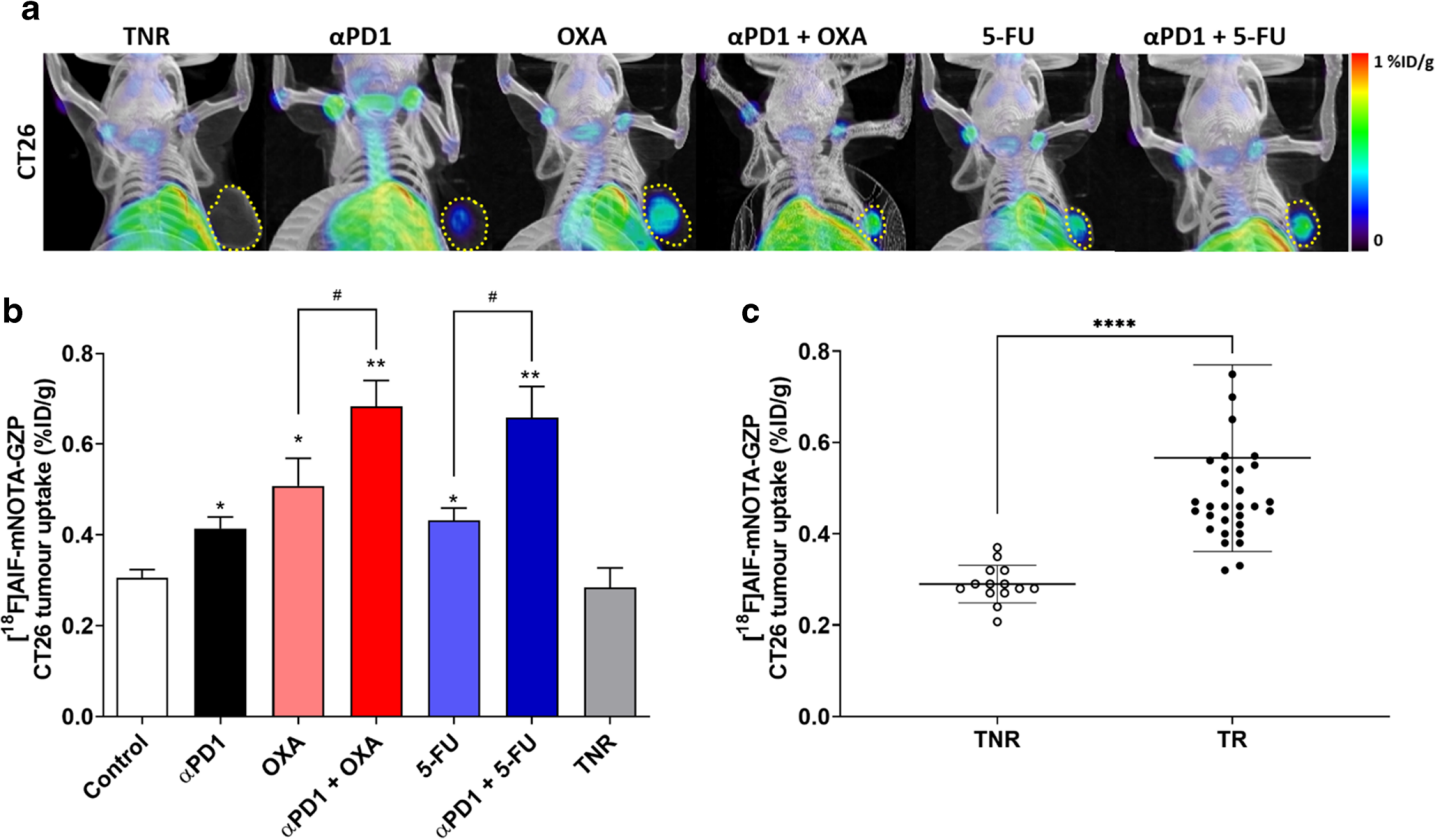
A. Representative maximum intensity projection PET/CT images of [^18^F]AlF-mNOTA-GZP tumor uptake in CT26 tumors: Treated non-responders (TNR), ⍺PD1 monotherapy, OXA monotherapy, combined ⍺PD1 + OXA, 5-FU monotherapy and combined ⍺PD1 + 5-FU treated animals. Yellow dashed line indicates tumor boundary. Mice administered [^18^F]AlF-mNOTA-GZP (~10 MBq intravenously), and images acquired from 60-80 mins post tracer injection. B. *In vivo* assessment of [^18^F]AlF-mNOTA-GZP tumor uptake from PET-CT defined volumes of interest (VOI) from individual mice subjected to ICI/ chemo/ ombination therapy. Significant increases in [^18^F]AlF-mNOTA-GZP tumor uptake was observed in treatment arms with ⍺PD-1, OXA, 5-FU, ⍺PD1 + OXA and ⍺PD1 + 5-FU when compared to treated non-responders (TNR, *n*=10 mice/ group; *P<0.05; **P<0.01 comparing TR to TNR and ^#^*P*<0.05 comparing chemo-ICI TR vs chemo alone; data shown as mean %ID/g ± S.E.M.). C. [^18^F]AlF-mNOTA-GZP tumor uptake in CT26 TRs and TNRs (****P<0.0001, data shown as individual %ID/g).

[^18^F]AlF-mNOTA-GZP PET imaging revealed adequate tumor-to-background contrast for tumor visualisation but showed heterogeneity of tumor’s tracer uptake *in vivo* across the different treatment arms (Fig. 3A) and was able to differentiate responders from treated non-responders (Table 1 and Fig. 3B and C) which was confirmed by *ex vivo* biodistribution (Supplementary Figure S1). Overall, low [^18^F]AlF-mNOTA-GZP uptake was observed in the control antibody treatment group and TNRs, and significantly higher uptake was observed in all TRs; ⍺PD1 (*P<0.05, *n*=7), OXA (*P<0.05, *n*=7), 5-FU (*P<0.05, *n*=7), combined ⍺PD1 + OXA (**P<0.01, *n*=9), and ⍺PD1 + 5-FU (**P<0.01, *n*=10, when compared to the TNR group, *n*=15). Furthermore, significantly higher uptake was observed in combination treatment groups ⍺PD1 + OXA (^#^*P*<0.05) and ⍺PD1 + 5-FU (^#^*P*<0.05) when compared to chemotherapy alone.

**Table 1.** [^18^F]AlF-mNOTA-GZP tumor uptake from PET-CT/MRI defined volumes of interest (VOI) from individual CT26 tumor-bearing mice subjected to chemotherapy and / or ICI treatment. Data are shown as mean %ID/g ± S.D. of control groups, treatment responders (TR) across individual treatment arms, and all treatment non-responders (TNR) (n=10 mice/ group; * *P*<0.05; ** *P*<0.01, comparing TR to TNR and ^#^*P*<0.05 comparing chemo-ICI TR vs chemo alone).

	[^18^F]AlF-*m*NOTA-GZP uptake in CT26 tumors
Control	0.31 ± 0.04
Treatment Responders (TR) αPD1	0.41 ± 0.06 *
OXA	0.51 ± 0.16 *
αPD1 + OXA	0.68 ± 0.15 **^,#^
5-FU	0.43 ± 0.08 *
αPD1 + 5-FU	0.66 ± 0.21 **^,#^
Treatment Non-Responders (TNR)	0.29 ± 0.04

### [^18^F]AlF-mNOTA-GZP Tumor Uptake Is Associated with GZB Expression in Tumor-Infiltrating Lymphocytes

**Fig. 4.**
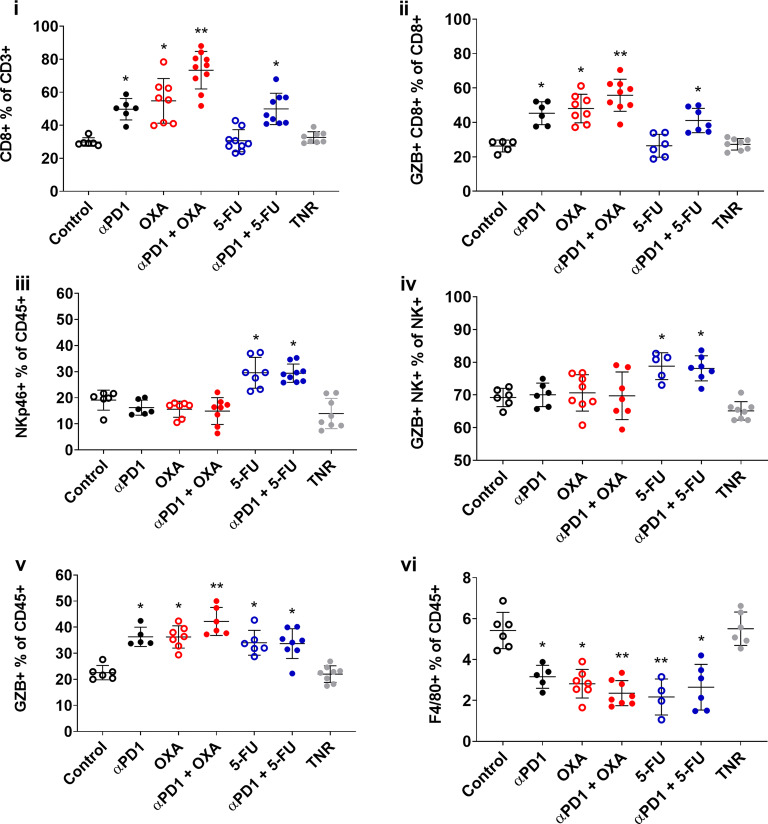
Multicolour flow cytometry analysis of immune cell profile of the tumor from CT26 tumor-bearing mice at day 14 post-induction of ICI monotherapy or combination therapies. Percentages of (i) CD8+ T cells relative to CD3+ cells, (ii) GZB+ CD8+ TILS relative to total CD8+ TILS, (iii) NK+ cells relative to total CD45+ cells (iv) GZB+ NK+ cells relative to total NK+ cells (v) GZB+ cells relative to CD45+ and (vi) F4/80+ relative to total CD45+ cells across all treatment arms. Data are shown as individual values with mean ± S.D. and are representative of n=5-10 mice/ group. * *P*<0.05; ** *P*<0.01 compared to TNR.

To quantify the changes in CT26 tumor-infiltrating lymphocytes (TILs) in response to therapy, we analyzed the distribution of different immune cell subsets of TRs to different therapeutic strategies and compared this to the TNR group (Fig. 4). t-SNE and Rphenoptype clustering were used to gain an overview on the immune cell populations and immunophenotypic changes across the different treatment arms. Rphenograph clustering identified CD8 T cells (clusters 1, 2, 11), CD4 T cells (clusters 6, 8, 17), NK cells (clusters 9, 15), CD11b myeloid cells (clusters 7, 12, 14), Ly6G-positive neutrophils (cluster 3), and SiglecF-positive eosinophils (cluster 10) (Fig. 3A). Separating the overall t-SNE into the different treatment arms revealed a striking increase of cluster 1 in OXA and αPD1+OXA treated groups and cluster 15 in 5-FU and αPD1+5-FU treated groups compared to the TNR arm, using the marker expression plots to annotate the Rphenotype-derived clusters (Supplementary Figures S2 and S3), cluster 1 and cluster 15 were identified as GZB+CD8+ T cells and GZB+NKp46+ NK cells (GZB+ NK+) respectively (Fig. 3A and B). Manual gating was performed on all tumors in order to quantify these differences in cell populations for statistical analysis (Supplementary Figure S4).

Many differences were observed when assessing the different immune cell populations between TR and TNRs (Supplementary Table S4). Significant differences were observed when assessing T cell populations, including significant changes in cell numbers associated with CD8+ T cells (CD8+ and GZB+ CD8+ T cells) and NK cells (NK+ and GZB+ NK+ cells) as well as GZB+ cells as a whole. Significant increases in CD8+ infiltration as a % of CD3+ cells were observed in responders to ⍺PD1 monotherapy (*P<0.05) and OXA monotherapy (**P<0.01) as well as ⍺PD1 + OXA combination therapy (***P<0.001) and ⍺PD1 + 5-FU combination therapy (*P<0.05) compared to TNRs. However, 5-FU monotherapy was not associated with increases in CD8+ cell infiltration (Table 2, Fig. 4Ci). Many of these infiltrating T cells also expressed granzyme B (GZB+). Significant increases in GZB+ CD8+ cell infiltration as a % of CD8+ cells were observed in responders to ⍺PD1 monotherapy (*P<0.05) and OXA monotherapy (**P<0.01) as well as ⍺PD1 + OXA combination therapy (***P<0.001) and ⍺PD1 + 5-FU combination therapy (*P<0.05) compared to TNRs. Again no significant increases in infiltrating GZB+ CD8+ infiltrating cells were observed in after treatment with 5-FU monotherapy (Table 2, Fig. 4Cii).

**Table 2.** FACS analysis of tumor-infiltrating leukocyte (TIL) populations from CT26 tumor-bearing mice at day 14 post-induction of αPD1 monotherapy, chemotherapy or combination therapies. Percentages of T cell subpopulations across control groups, treatment responder (TR) arms, and all treatment non-responders (TNR) across all treatment arms. Data are shown as mean % of cells ± S.D. and are representative of *n*=5–10 mice/group, * *P*<0.05; **P<0.01, comparing TR to TNR.

	Immune cell subsets associated with CT26 tumors					
	CD8+ % of CD3+	GZB+ CD8+ % of CD8+	NK+ % of CD45+	GZB+ NK+ % of NK+	GZB+ % of CD3+	F4/80+ % of CD45+
Control	30.11 ± 2.81	26.28 ± 3.43	19.09 ± 3.83	69.21 ± 2.79	22.57 ± 2.75	5.42 ± 0.89
TR αPD1	51.82 ± 4.36*	46.91 ± 6.13*	16.52 ± 3.11	70.89 ± 3.23	36.29 ± 3.69*	3.16 ± 0.56*
OXA	54.79 ± 13.49*	48.06 ± 8.30*	15.61 ± 3.08	70.62 ± 5.55	36.80 ± 4.38*	2.82 ± 0.70*
αPD1 + OXA	73.35 ± 11.37**	55.77 ± 9.35**	14.90 ± 5.14	69.71 ± 7.28	42.18 ± 5.38**	2.35 ± 0.61**
5-FU	30.65 ± 6.69	26.41 ± 6.64	29.56 ± 5.91*	78.77 ± 4.10*	34.01 ± 4.77*	2.17 ± 0.88**
αPD1 + 5-FU	49.92 ± 9.48*	41.12 ± 7.05*	29.40 ± 3.51*	78.10 ± 3.84*	33.67 ± 5.71*	2.65 ± 1.12*
TNR	33.37 ± 3.88	28.38 ± 4.65	14.86 ± 5.15	65.18 ± 2.64	22.00 ± 3.18	5.51 ± 0.82

Significant increases in NK+ cell infiltration as a % of CD45+ cells were observed only in responders to 5-FU monotherapy (*P<0.05) and ⍺PD1 + 5-FU combination therapy (*P<0.05) compared to TNRs (Table 2, Fig. 4Ciii). No significant increases were observed in the other groups. Many of these infiltrating NK cells also expressed granzyme B (GZB+), with significant increases in GZB+ NK+ cell infiltration as a % of NK+ cells observed in responders to 5-FU monotherapy (*P<0.05) and ⍺PD1 + 5-FU combination therapy (*P<0.05) compared to TNRs (Table 2, Fig. 4Civ). Again, no significant increases were observed in the other groups. Overall, the data shows that in all cases successful response to therapy was associated with significant increases in GZB+ cell infiltration. As shown in Table 2, GZB+ cell infiltration as a % of CD45+ cells were observed in all responding groups; ⍺PD1 monotherapy (*P<0.05), OXA monotherapy (*P<0.05), 5-FU monotherapy (*P<0.05) as well as ⍺PD1 + OXA combination therapy (**P<0.01) and ⍺PD1 + 5-FU combination therapy (*P<0.05) compared to TNRs (Table 2, Fig. 4Cv). Tumor responders not only increased GZB+ cell infiltration but also reduced infiltration of cell types associated with suppression/resistance, decreases in F4/80+ cells were observed in all responding groups: ⍺PD1 monotherapy (*P<0.05), OXA monotherapy (*P<0.05), ⍺PD1 + OXA combination therapy (**P<0.01), 5-FU monotherapy (**P<0.01) and ⍺PD1 + 5-FU combination therapy (*P<0.05) compared to TNRs (Table 2, Fig. 4Cvi).

## Discussion

Chemotherapy response is classically defined by evaluating morphologic changes in tumor volume defined by CT or MRI following RECIST criteria [22]. However, tumors treated with ICIs may remain stable or even increase in size before ultimately responding to therapy [23]. This divergence adds complexity for on-going clinical trials attempting to quantify tumor responsiveness to ICIs when combined with chemotherapy [24]. The expectation is that tumors will respond better to the combination than to monotherapy; however, often combination therapy is no more effective than successful monotherapy, administering multiple drugs simply increases the chance of experiencing a meaningful anti-tumor response to any single drug in the combination [25]. While tumor growth inhibition (TGI) may provide a simple way to determine the rate of response to a therapy, it provides no information on whether therapies are working alone or synergistically.

Both 5-FU and OXA are well-known to function as effective chemotherapeutic adjuvants inhibiting tumor cell proliferation; however, they can also exert immunomodulatory effects in numerous ways. 5-FU can facilitate antigen uptake by dendritic cells (DCs) and selectively kills monocyte-derived suppressor cells (MDSCs) while sparing other lymphocyte subtypes [9, 10]. OXA upregulates PD-L1 expression on tumors and DCs and can induce immunogenic cell death (ICD), a form of apoptotic cell death associated with the release of damage-associated molecular patterns (DAMPs) [26]. These DAMPs, in combination with cancer antigens, induce maturation of dendritic cells and can lead to an adaptive immune response against tumor cells [27–29]. The immune effects for both 5-FU and OXA are observed at lower doses than typically used clinically, the doses chosen in the current study were designed to mimic this, the equivalent dose in humans has been shown to be minimally symptomatic [30] and in the animals displayed no side effects associated with high-dose chemotherapy. The effect of chemotherapy-induced changes in the immune environment and how they affect ICIs when given in combination is difficult to quantify using the standard measure of tumor growth inhibition.

Non-invasive molecular imaging with radiolabelled GZB-targeting peptides has been shown to stratify response to ICIs administered alone or in combination [14, 15, 20, 31]. The current study, however, is the first to show that GZB targeting peptides can stratify response to chemotherapies that exert an immunostimulatory effect either alone or in combination with ICIs. Tumor uptake of [^18^F]AlF-mNOTA-GZP was well correlated to tumor growth inhibition across the treatment arms and when separated by treatment response, uptake of [^18^F]AlF-mNOTA-GZP was significantly higher in PD1, OXA and 5-FU responsive tumors compared to TNRs (Table 1, Fig. 3B). Tumors responsive to chemo-ICI combinations PD1+OXA or PD1+5-FU showed even greater uptake than those treated with mono therapy alone (Table 1, Fig. 3B, ^#^*P*>0.05). This additive effect may be caused by the recruitment of different immune cell types. Tumors that responded to OXA monotherapy showed significant increases in CD8+ and CD8+GZB+ TILs and decreases in F4/80 myeloid cells, a similar immune cell profile observed in responders to αPD1 monotherapy (Table 2, Fig. 4). Tumors that responded to combined OXA and αPD1 therapy showed even greater increases in CD8+ and CD8+GZB+ TILs, a profile mirrored by increases in tumor retention of [^18^F]AlF-mNOTA-GZP. 5-FU treatment, however, worked via a different mechanism of action, inducing no change in CD8+ TILs but instead a significant increase in tumor infiltrating NK+ and GZB+ NK+ cells. When administered as a combination, 5-FU and αPD1 showed no additive effect on NK+ cell infiltration (Table 2, Fig. 4) but still showed increases in tumor retention of [^18^F]AlF-mNOTA-GZP. The data indicate that in both cases response to therapy is driven by GZB+ cell infiltration, increases in GZB+ CD8+ TILs caused by both parts of the therapy when OXA and PD1 are combined and increases in GZB+ NK+ cells and GZB+ CD8+ cells independently induced by 5-FU and PD1 when used in combination. Further studies will be needed to determine whether stratification of tumor response is possible using other chemo-ICI combinations.

Much work is needed to determine whether the preclinical data outlined in the current study are clinically translatable. Previous studies have shown that a simple amino acid substitution (IEFD to IEPD) confers selectivity for human Granzyme B [20] versus other granzymes; however, it is not known whether IEPD is selective for Granzyme B over caspases. Typically, clinical assessment of therapy response is based on tumor size reduction, whether granzyme B imaging peptides will be able to distinguish between progressive disease and partial response is difficult to determine from studies based on tumor growth inhibition. Capsases, which also cut peptides with Asp/Glu at position 1, may be released when tumors debulk rapidly due to therapy response or necrosis and this may further complicate clinical assessment of these imaging peptides. In the current study, the tumor to blood ratio in TRs is >1.5 and the tumor to muscle ratio is >1.5 primarily due to rapid clearance from the blood; however, the overall tumor uptake is still low. Low tumor uptake will hamper the utility of granzyme B peptides in tumors located in organs with higher background such as those organs associated with peptide excretion including the bladder and kidneys and to a lesser extent the liver and intestines. Furthermore, granzyme B targeting peptides ability to stratify response has been shown to be affected by the background immune phenotype [15], patient tumors tend to be large and heterogeneous, with areas of necrosis introducing variability that may complicate clinical assessment.

Overall, the preclinical data in the current study suggests that while the immunomodulatory mode of action of the chemotherapies may be different, they both induce tumor lysis in part through the release of granzyme B and that the detection of this granzyme B release acts as biomarker for efficacy in the syngeneic tumors studied. If granzyme B targeting peptides can be successfully translated to the clinic they may provide information on the efficacy of chemotherapy-ICI combinations aiding in trials striving to enhance responsiveness to ICIs.

## Supplementary Information

ESM 1 (DOCX 2881 kb)
