# Supplementary material for: Granzyme B PET Imaging of Combined Chemotherapy and Immune Checkpoint Inhibitor Therapy in Colon Cancer
Source: Mol Imaging Biol. 2021 Mar 12;23(5):714–23. doi: 10.1007/s11307-021-01596-y (PMC8410722; doi:10.1007/s11307-021-01596-y)
Supplement: Supplementary file 1 — (DOCX 2881 kb) [file 11307_2021_1596_MOESM1_ESM.docx]

# SUPPLEMENTARY MATERIALS

**Granzyme B PET imaging of combined chemotherapy and immune checkpoint inhibitor therapy in colon cancer.**

Julian L Goggi^a*^; Siddesh V Hartimath^a^; Tan Yun Xuan^a^; Shivashankar Khanapur^a^; Beverly Jieu^d^; Hui Xian Chin^b^; Boominathan Ramasamy^a^; Peter Cheng^a^; Tang Jun Rong^a^; Yong Fui Fong^a^; Tsz Ying Yuen^d^; Rasha Msallam^e^; Ann-Marie Chacko^e^; Laurent Renia^b^; Charles Johannes^c^, You Yi Hwang^b^; Edward G Robins^a,f^.

^a^ Singapore Bioimaging Consortium, Agency for Science, Technology and Research (A*STAR), 11 Biopolis Way, #01-02 Helios, Singapore, 138667

^b^ Singapore Immunology Network, A*STAR, 8A Biomedical Grove, Immunos, Singapore, 138648

^c^ p53 Laboratory, A*STAR, 8A Biomedical Grove, #06-04/05, Neuros/Immunos, Singapore 138665

^d^ Institute of Chemical and Engineering Sciences (ICES), A*STAR, 8 Biomedical Grove, #07, Neuros, Singapore 138665

^e^ Laboratory for Translational and Molecular Imaging (LTMI), Cancer and Stem Cell Biology Programme, Duke-NUS Medical School, 8 College Road, Singapore 169857

^f^ Clinical Imaging Research Centre (CIRC), Yong Loo Lin School of Medicine, National University of Singapore, Singapore, 117599

*Corresponding author:

Dr Julian Luke Goggi

Julian_goggi[@sbic.a-star.edu.sg](mailto:@sbic.a-star.edu.sg)

Tel: +65 6478 8901

Fax: +65 6478 8908

| **Treatment arm** | **Days post inoculation** | **CT26 tumour volume**  **(mm^3^ ± SD)** |
| --- | --- | --- |
| **Control** | 6  9  12  15  19  21 | 115.9 ± 33.2  175.6 ± 47.0  277.6 ± 66.0  466.8 ± 107.1  972.3 ± 225.8  1440.4 ± 294.0 |
| **Treatment Responders (TR)**  **αPD1** | 6  9  12  15  19  21 | 115.8 + 23.7  158.8 + 24.2  232.8 + 72.2  313.6 + 66.0  412.8 + 109.6  498.8 + 143.6 |
| **OXA** | 6  9  12  15  19  21 | 136.9 ± 26.5  150.0 ± 42.1  188.6 + 25.6  222.9 ± 41.1  267.9 ± 110.6  339.3 ± 164.7 |
| **αPD1 + OXA** | 6  9  12  15  19  21 | 128.2 ± 27.6  110.7 ± 34.1  136.6 ± 53.7  136.3 ± 55.2  160.4 ± 108.0  196.7 ± 153.7 |
| **5-FU** | 6  9  12  15  19  21 | 143.5 ± 27.7  150.2 ± 31.5  156.8 ± 49.2  181.8 ± 76.0  223.9 ± 126.6  270.7 ± 157.5 |
| **αPD1 + 5-FU** | 6  9  12  15  19  21 | 132.0 ± 22.2  132.9 ± 21.2  167.2 ± 41.5  200.1 ± 66.3  173.5 ± 33.9  202.9 ± 41.3 |
| **Treatment Non-Responders (TNR)** | 6  9  12  15  19  21 | 136.2 + 30.6  209.3 + 50.5  310.1 + 60.7  516.7 + 140.8  760.9 + 130.7  1040.8 + 168.8 |

**Supplementary Table S1.** Summary of tumour volumes in controls, ICI and or chemotherapy treatment responders (TR) and treatment non-responders (TNR) across all therapy arms in the syngeneic CT26 colon cancer model

|  | **Treatment Responders**  **/**  **Treatment Arm**  **(No. mice)** |
| --- | --- |
| **ICI Treatment** | **CT26** |
| Control | 0/10 |
| αPD1 | 7/15 |
| OXA | 7/10 |
| αPD1 + OXA | 9/10 |
| 5-FU | 7/10 |
| αPD1 + 5-FU | 10/10 |
| % Monotherapy Response | 60.0 |
| % Combined Therapy Response | 95.0 |
| % Overall Therapy Response | 72.7 |

Supplementary Table S2. Summary of ICI and/ or chemotherapy treatment responders (TR) per therapy arm in the syngeneic CT26 colon cancer model

| **Treatment arm** | **%TGI (mean ± SD)** |
| --- | --- |
| αPD1 | 51.9 ± 7.7 |
| OXA | 77.6 ± 15.2 |
| αPD1 + OXA | 82.3 ± 19.8 |
| 5-FU | 74.5 ± 11.4 |
| αPD1 + 5FU | 92.2 ± 7.9 |
| TNR | 21.5 ± 14.1 |

**Supplementary Table S3.** Tumour growth inhibition % on day 21 for each treatment arm compared to control (TNR, treated non-responder).

**A**

|  | **CD8+ %**  **of CD3+** | **GZB+ CD8+ %**  **of CD8+** | **NK+ %**  **of CD45+** | **GZB+ NK+ %**  **of NK+** | **GZB+ %**  **of CD3+** | **F4/80+ %**  **of CD45+** |
| --- | --- | --- | --- | --- | --- | --- |
| **Control** | 30.11 ± 2.81 | 26.28 ± 3.43 | 19.09 ± 3.83 | 69.21 ± 2.79 | 22.57 ± 2.75 | 5.42 ± 0.89 |
| **TR**  **αPD1** | 51.82 ± 4.36* | 46.91 ± 6.13* | 16.52 ± 3.11 | 70.89 ± 3.23 | 36.29 ± 3.69* | 3.16 ± 0.56* |
| **OXA** | 54.79 ± 13.49* | 48.06 ± 8.30* | 15.61 ± 3.08 | 70.62 ± 5.55 | 36.80 ± 4.38* | 2.82 ± 0.70* |
| **αPD1 + OXA** | 73.35 ± 11.37** | 55.77 ± 9.35** | 14.90 ± 5.14 | 69.71 ± 7.28 | 42.18 ± 5.38** | 2.35 ± 0.61** |
| **5-FU** | 30.65 ± 6.69 | 26.41 ± 6.64 | 29.56 ± 5.91* | 78.77 ± 4.10* | 34.01 ± 4.77* | 2.17 ± 0.88** |
| **αPD1 + 5-FU** | 49.92 ± 9.48* | 41.12 ± 7.05* | 29.40 ± 3.51* | 78.10 ± 3.84* | 33.67 ± 5.71* | 2.65 ± 1.12* |
| **TNR** | 33.37 ± 3.88 | 28.38 ± 4.65 | 14.86 ± 5.15 | 65.18 ± 2.64 | 22.00 ± 3.18 | 5.51 ± 0.82 |

**B**

|  | **CD3+ %**  **of CD45+** | **CD4+ %**  **of CD3+** | **CD25+ CD4+ %**  **of CD4+** | **CD4+ Teff %**  **of CD4+** | **CD4+ Treg %**  **of CD4+** | **Eos %**  **of CD45+** |
| --- | --- | --- | --- | --- | --- | --- |
| **Control** | 43.07 ± 8.48 | 42.98 ± 10.42 | 40.56 ± 2.48 | 62.10 ± 12.60 | 29.16 ± 8.46 | 1.57 ± 0.62 |
| **TR**  **αPD1** | 40.74 ± 5.28 | 44.02 ± 10.78 | 42.87 ± 7.40 | 60.44 ± 6.30 | 31.92 ± 5.76 | 1.61 ± 1.01 |
| **OXA** | 46.81 ± 14.55 | 33.73 ± 10.21* | 39.47 ± 4.44 | 72.60 ± 6.48 | 22.80 ± 10.09 | 1.84 ± 1.04 |
| **αPD1 + OXA** | 48.83 ± 5.92 | 25.61 ± 9.29* | 39.72 ± 4.44 | 71.30 ± 10.43 | 24.06 ± 5.43 | 2.08 ± 0.88 |
| **5-FU** | 44.15 ± 16.76 | 48.67 ± 7.62 | 46.24 ± 4.90 | 57.83 ± 8.07 | 30.78 ± 4.61 | 2.39 ± 1.18 |
| **αPD1 + 5-FU** | 44.07 ± 6.77 | 52.67 ± 15.16 | 47.27 ± 3.01 | 55.17 ± 5.39 | 37.76 ± 6.27 | 3.26 ± 1.67 |
| **TNR** | 41.09 ± 7.42 | 52.67 ± 15.16 | 42.86 ± 1.48 | 58.06 ± 12.47 | 32.35 ± 6.53 | 1.61 ± 0.52 |

**C**

|  | **CD25+ CD8+ %**  **of CD8+** | **Perforin + CD8+ %**  **of CD8+** | **gMDSC+ %**  **of F4/80+** | **mMDSC+ NK+ %**  **of F4/80+** | **Perforin+ NK+ %**  **of NK+** |
| --- | --- | --- | --- | --- | --- |
| **Control** | 5.53 ± 1.54 | 43.94 ± 10.90 | 6.45 ± 5.99 | 9.38 ± 1.57 | 88.16 ± 1.46 |
| **TR**  **αPD1** | 4.57 ± 1.62 | 69.23 ± 3.93 | 6.57 ± 3.24 | 7.23 ± 4.91 | 92.95 ± 3.43 |
| **OXA** | 6.28 ± 3.62 | 77.91 ± 13.08* | 8.20 ± 2.50 | 8.94 ± 6.82 | 93.64 ± 3.95 |
| **αPD1 + OXA** | 4.42 ± 2.49 | 77.79 ± 8.20* | 9.05 ± 4.32 | 5.98 ± 2.69 | 90.85 ± 6.79 |
| **5-FU** | 6.29 ± 2.61 | 50.73 ± 17.35 | 6.26 ± 1.99 | 6.63 ± 3.88 | 92.26 ± 3.72 |
| **αPD1 + 5-FU** | 7.67 ± 4.21 | 59.15 ± 17.63 | 5.97 ± 2.57 | 5.02 ± 3.44 | 93.53 ± 4.01 |
| **TNR** | 7.64 ± 3.17 | 48.16 ± 8.76 | 6.80 ± 1.60 | 8.55 ± 2.60 | 89.18 ± 4.99 |

**Supplementary Table S4.** Table showing the tumour associated immune cell populations from CT26 tumour-bearing mice at day 14 post-induction of αPD1 monotherapy, chemotherapy or combination therapies. **A**. Percentages of CD8+, GZB+ CD8+, NK+, GZB+ NK+, GZB+ and F4/80+ immune cell subpopulations are shown across control groups, treatment responders (TR) and treatment non-responders (TNR) across all treatment arms. **B**. Percentages of CD3+, CD4+, CD25+ CD4+, CD4+ Teff, CD4+ Treg and Eos+ immune cell subpopulations are shown across control groups, treatment responders (TR) and treatment non-responders (TNR) across all treatment arms. **C**. Percentages of CD25+ CD8+, perforin+ CD8+, gMDSC+, mMDSC+ and perforin+ NK+ immune cell subpopulations are shown across control groups, treatment responders (TR) and treatment non-responders (TNR) across all treatment arms. Data are shown as mean % of cells ± S.D. and are representative of n=5-10 mice/ group, * *P*<0.05; ** *P*<0.01, *** *P*<0.001, comparing TR to TNR.

**
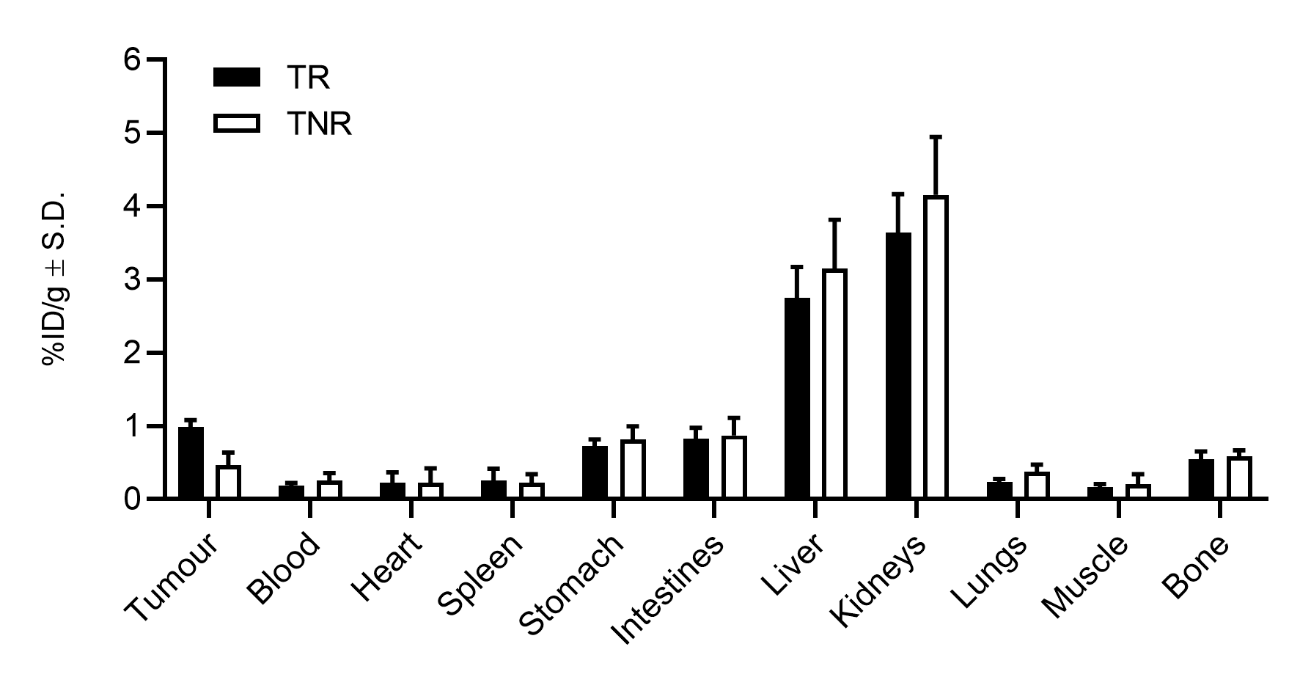
**

**Supplementary Figure S1.** Ex vivo biodistribution analysis of [^18^F]AlF-mNOTA-GZP retention in selected organs. Chemotherapy-ICI treated responder (TR, black) and treated non responder (TNR, white) animals were sacrificed 80min post-injection, tissues excised, weighed and radioactivity quantified using a Wallac gamma counter. Bars represent the mean of 5 animals ± SEM.

**
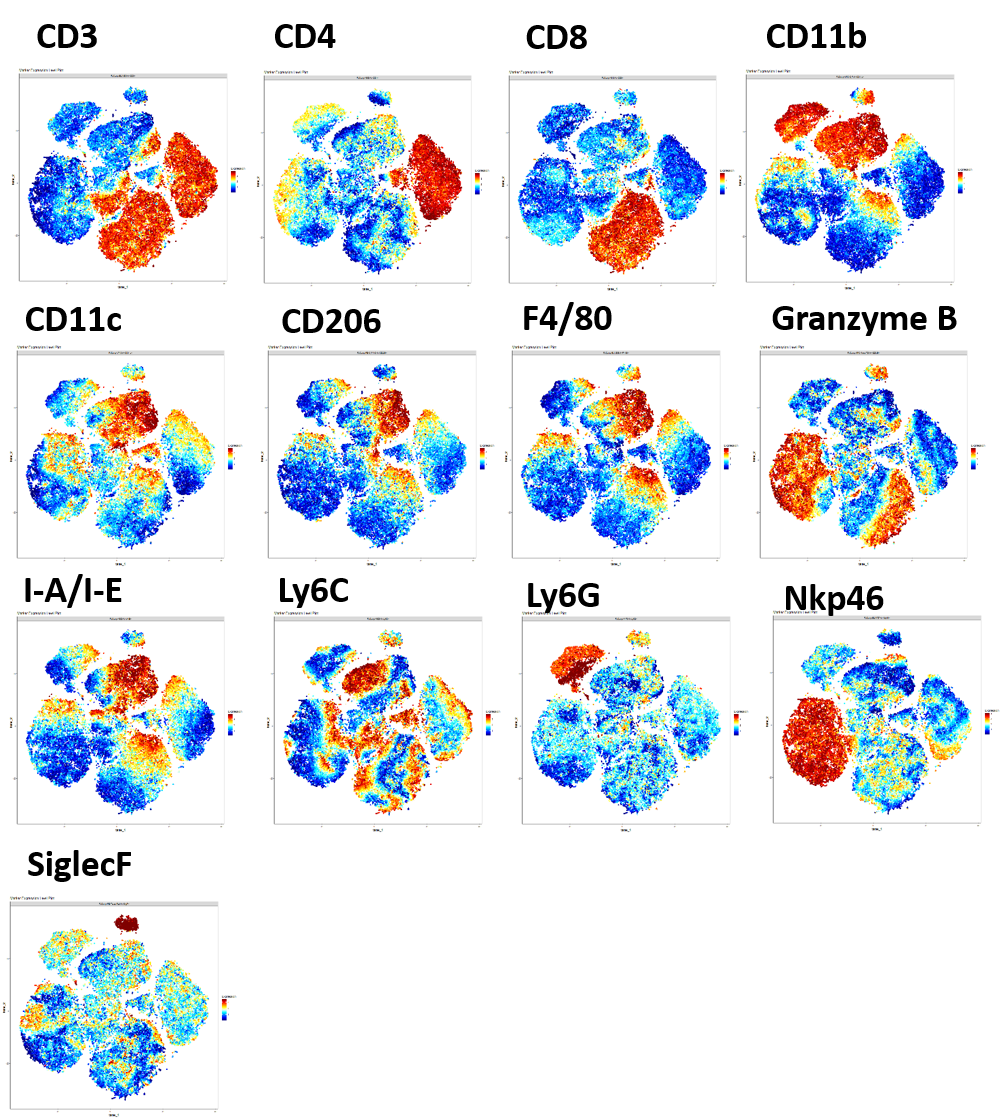
**

**Supplementary Figure 2.** Marker expression level plots of the markers used for Rphenograph clustering on all tumours used for the t-SNE.

**
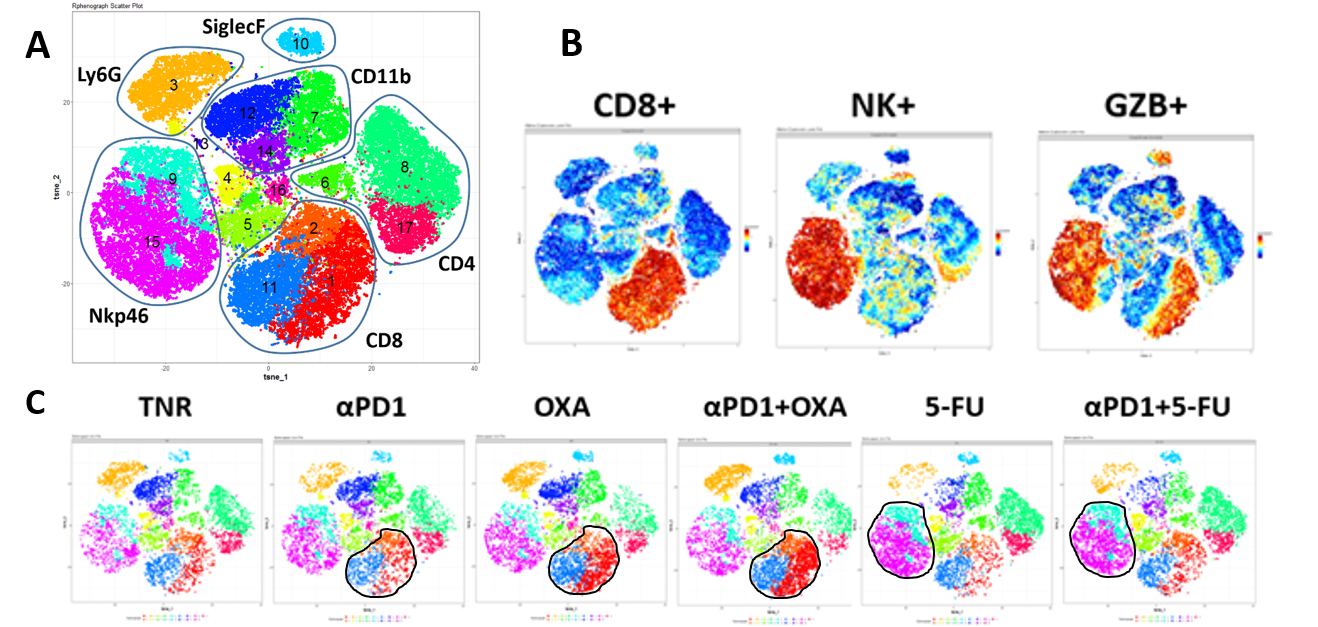
**

**Supplementary Figure 3.** Multicolour Flow cytometry analysis of immune cell profile of the tumour from CT26 tumour-bearing mice at day 14 post-induction of ICI monotherapy or combination therapies. **A.** t-SNE plot showing unbiased Rphenograph clustering of cell populations based on the expression of CD3, CD4, CD8, CD11b, CD11c, CD206, F4/80, Granzyme B, I-A/I-E, Ly6C, Ly6G, Nkp46 and Siglec-F.  **B**. t-SNE plots identifying the Rphenograph clusters expressing CD8 (left), NKp46 (middle), Granzyme B (right). **C.** tSNE plots showing the distribution of cells in the Rphenograph clusters in each treatment arm. TNR n=6, αPD1 n=6, OXA n=7, αPD1+OXA n=7, 5-FU n=7, αPD1+5-FU n=7 (clusters showing significant changes ringed in black).


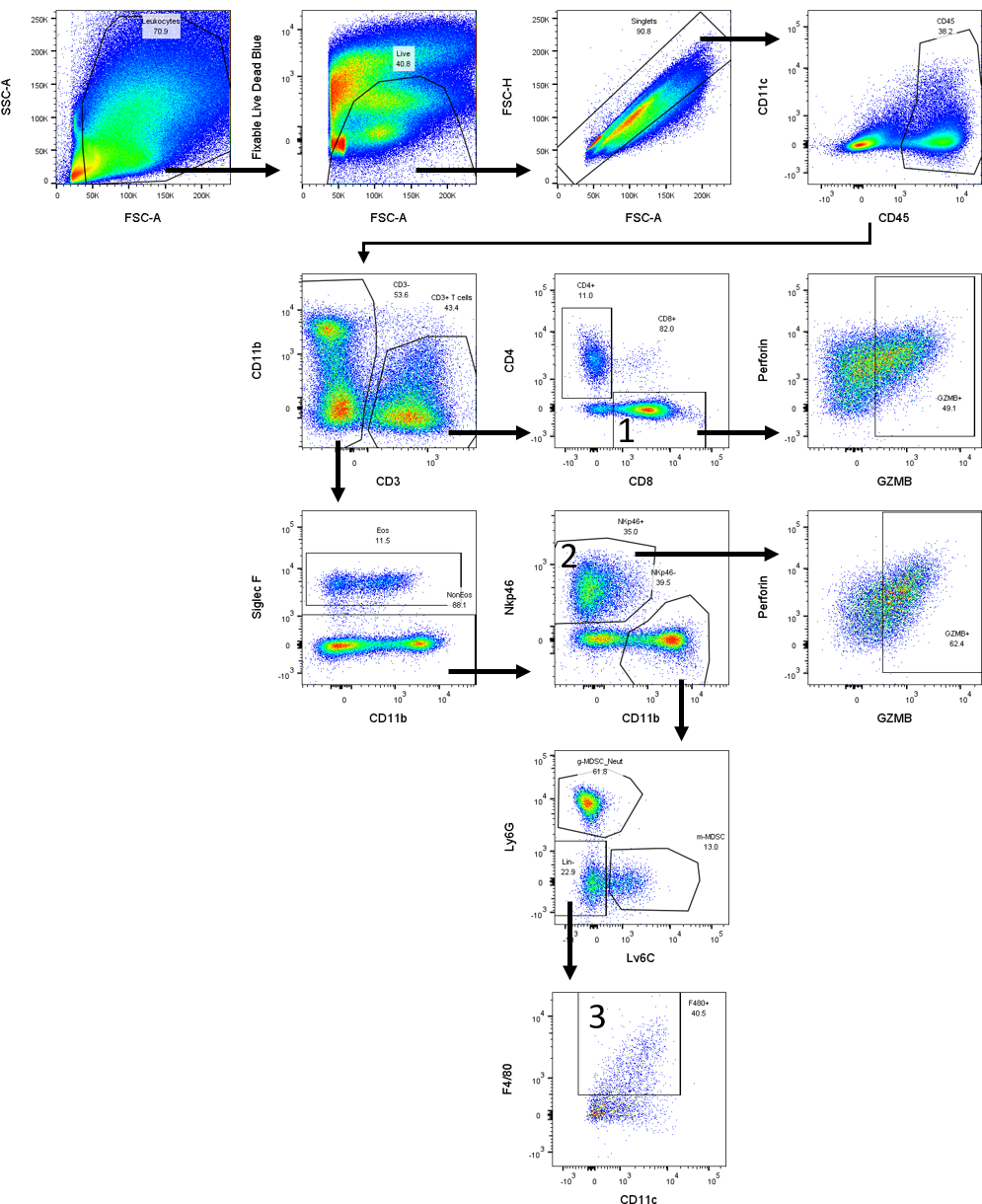


**Supplementary Figure 4.** Representative manual gating strategy for immunophenotyping of tumour samples. Viable hematopoietic cells were identified by size, viability stain-negative, singlet gating and CD45-positive expression. CD3+CD8+ T cells (1), CD3-SiglecF-Nkp46+ NK cells (2) were identified and investigated for Granzyme B expression. F4/80 cells (3) were identified as CD3-SiglecF-NKp46-Ly6G-Ly6c-F4/80.
